# Green synthesis of AuNPs using *Cistanche tubulosa* extract and their broad-spectrum antimicrobial, antiparasitic, and scolicidal activities

**DOI:** 10.1039/d5ra08159a

**Published:** 2025-12-15

**Authors:** Hanieh Karimi, S. Ebrahim Seifati, Damoun Razmjoue, Soraya Ghayempour

**Affiliations:** a Department of Arid Land and Desert Management, School of Natural Resources and Desert Studies, Yazd University Yazd Iran seifati@yazd.ac.ir; b Department of Pharmacology, Faculty of Medicine, Yasuj University of Medical Sciences Yasuj Iran; c Department of Textile Engineering, Faculty of Engineering, Yazd University Yazd Iran

## Abstract

The green synthesis of metal nanoparticles has emerged as a promising alternative to traditional antimicrobial agents due to the simultaneous effects of metal nanoparticles and herbal products on antimicrobial properties. In this study, we present the one-step green synthesis of gold nanoparticles (AuNPs) using *Cistanche tubulosa* (*C. tubulosa*), a medicinal plant known for its diverse therapeutic properties, including antioxidant, anti-inflammatory, and antimicrobial effects. The synthesis was carried out by mixing the aqueous extract of *C. tubulosa* with tetrachloroauric acid (HAuCl_4_), resulting in the reduction of gold ions and the formation of AuNPs. The time of 70 min was selected as the optimum reaction time by evaluating the absorption peak at 545 nm in UV-vis spectra. Scanning electron microscopy (SEM) and transmission electron microscopy (TEM) images indicated AuNPs were successfully synthesized with quasi-spherical morphology in the particle size range of 20–70 nm. The synthesized AuNPs exhibited significant antibacterial activity against both Gram-positive and Gram-negative bacteria, including *Staphylococcus aureus*, *Escherichia coli*, and *Bacillus cereus*, with the best antibacterial activity being for *Bacillus cereus* with as low as a minimum inhibitory concentration (MIC) of 37.50 µg mL^−1^. Furthermore, the AuNPs demonstrated potent antiparasitic activity against *Giardia lamblia*, a common protozoan parasite, with 100% inhibition at 120 µg mL^−1^ for 480 minutes. The nanoparticles also exhibited excellent scolicidal activity against *Echinococcus granulosus* protoscolices, with 100% inhibition at 180 and 60 µg per mL AuNPs for 30 and 60 minutes, respectively. These findings indicate the promising potential of *C. tubulosa*-synthesized AuNPs as a solution for combating a wide range of infectious and parasitic diseases and an attractive alternative to conventional antibiotics and antiparasitic agents. This study lays the foundation for future research into the *in vivo* efficacy and clinical application of these green-synthesized gold nanoparticles in the treatment of infectious diseases.

## Abstract 2

In this study, *Cistanche tubulosa* extract was used as the natural stabilizing and reducing agent in synthesis of AuNPs with good antimicrobial, antiparasitic, and scolicidal activities.

## Graphical abstract

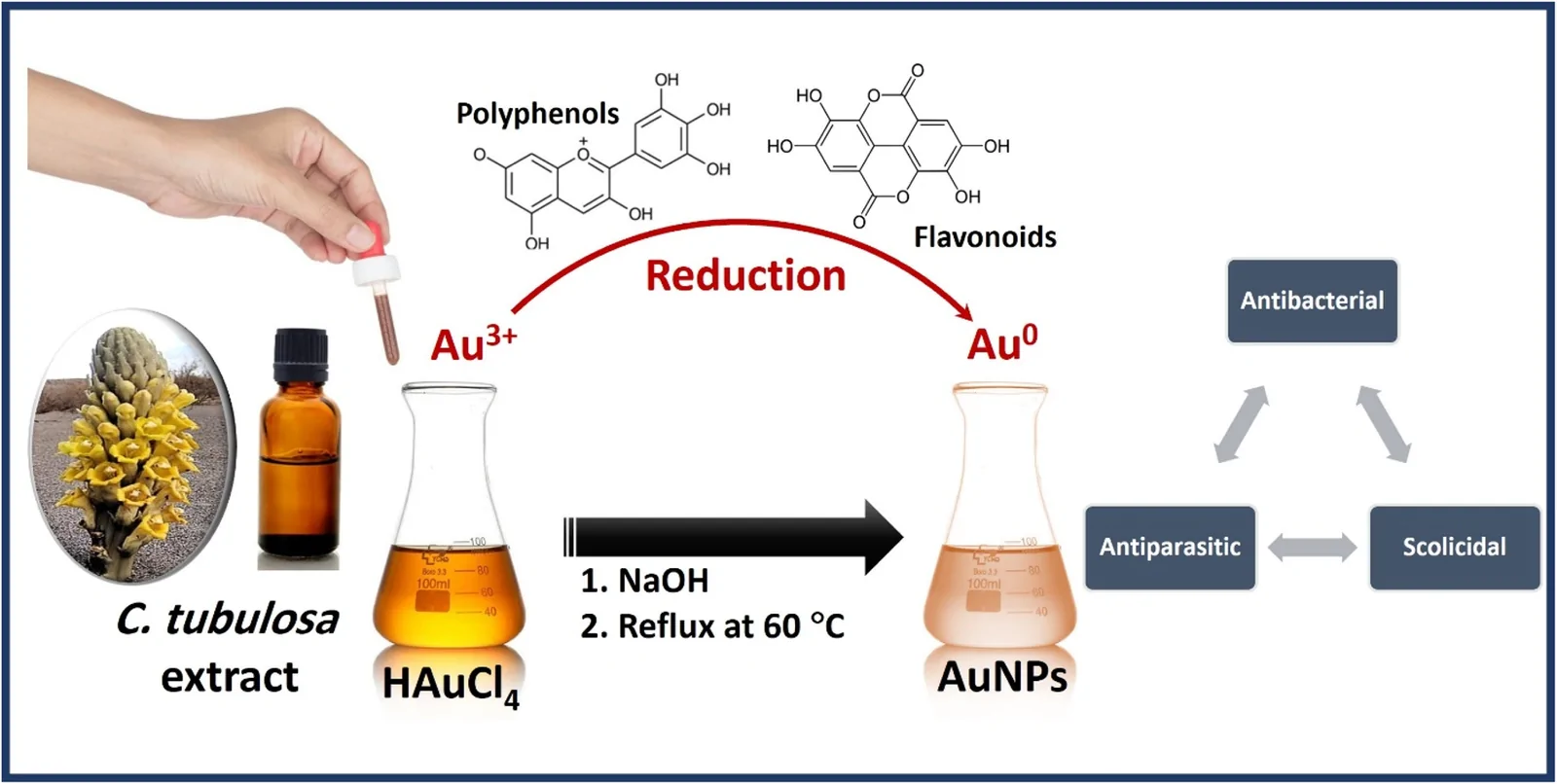

## Introduction

Today, in the field of healthcare and public health, countries are faced with a wide range of infectious pathogens (different genera and species of bacteria, fungi, viruses, and parasites), and this has led to the use of various ranges of antibiotics. However, on the other hand, these microorganisms have become resistant to some of these antibiotics, which has reduced their therapeutic effectiveness against these microorganisms. Therefore, given the existence of such problems (drug resistance) in the field of combating pathogens, it is necessary to study and investigate some new compounds of plant origin that have appropriate efficacy for controlling this group of microorganisms and ultimately to understand their resistance.^1–6^

The parasitic disease giardiasis is one of the most common infectious diseases in developing countries, with symptoms of acute diarrhea, bloating, and abdominal cramps, affecting about 280 million people annually. The causative agent of this parasitic infectious disease is the flagellated protozoan parasite *Giardia lamblia*. Common medications for the treatment of this disease have several side effects, including nausea, a metallic taste in the mouth, and psychosis.^7,8^ Another common disease in developing countries is liver hydatid cyst disease, which is a parasitic infectious disease common to humans and livestock. The main cause of this disease is the parasite *Echinococcus granulosus*, which in its larval stage causes hydatid cysts in the liver and lungs. The drugs prescribed for these patients have shown severe side effects such as leukopenia, hepatotoxicity alopecia, and thrombocytopenia. Considering the complications of drug treatment, surgery, and reducing the risk of shedding cyst contents (protoscolex) during surgery, it is necessary to use natural compounds or substances that can be considered a natural scolicidal agent.^9–11^

In recent years, due to the wealth of resources, green synthesis has been applied as a low-cost and eco-friendly approach to develop advanced nanostructures.^12–14^ Metal nanoparticles synthesized and produced by green methods based on plant extracts can be considered antimicrobial agents. The presence of phytochemicals such as flavonoids, tannins, ketones, proteins, aldehydes, and carboxylic acids in plant extracts acts as reducing agents in the synthesis of metal nanoparticles.^15–20^ Among them, gold nanoparticles are a good candidate in various biomedicine studies. Gold nanoparticles biosynthesized using plant products have been indicated diverse biological properties such as antibacterial, antifungal, antiparasitic, antioxidant, antidiabetic, anticoagulant, catalytic, and non-toxic activities.^21–25^

*Cistanche*, a member of the Orobanchaceae family and commonly referred to as “Ginseng of the Deserts”, thrives in arid and semi-arid regions, particularly in deserts of northwestern China, southern Russia, southern Mongolia, and coastal areas of the Persian Gulf, as well as saline sandy plains in India and Iran. It has been used for centuries in traditional Chinese medicine as a tonic. The most prevalent species worldwide and in Iran is *Cistanche tubulosa*, which adapts to various ecological conditions, including dry habitats and coastal areas, and grows in saline, clayey, and alkaline soils.^26–28^ This plant is rich in bioactive compounds such as phenylethanoid glycosides (cistanoside A, B, echinacosides, acteosides, isoacteosides, and others), iridoids, terpenes, and lignans, with phenylethanoid glycosides being particularly noted for their antioxidant, anticancer, neuroprotective, anti-aging, anti-inflammatory, antifungal, and antibacterial properties.^29–32^

According to our studies, despite the valuable health benefits of this plant and the presence of diverse valuable and medicinal compounds, no research has been conducted on the application of this plant's products in the green synthesis of metal nanoparticles. The compounds present in the *C. tubulosa* extract, such as flavonoids, polyphenols, and amino acids, can play an important role in the synthesis of AUNPs by reducing Au ions to neutral gold and stabilizing the resulting AuNPs. In this study, for the first time, the aqueous extract of the aerial parts of this medicinal plant was used to synthesize gold nanoparticles. After characterizing the synthesized nanoparticles, their antibacterial, antiparasitic, and scolicidal properties were investigated.

## Experimental

### Chemicals

The aerial parts of *C. tubulosa* were collected from the fields of Yazd Province, Iran. Tetrachloroauric(iii) acid trihydrate (HAuCl_4_·3H_2_O, 99%), Mueller–Hinton agar, and Muller–Hinton broth were received from Merck Co. Eosin and Gentamicin antibiotic were purchased from Sigma-Aldrich. The reagents used were of the highest analytical grade available. All solutions were prepared using deionized water.

### Preparation of *C. tubulosa* extract

The aerial parts of *C. tubulosa* were ground and dried for 20 days in the shade. To prepare the aqueous extract, 10 g of the plant powder was added to 200 mL of sterilized deionized water and heated for 20 minutes at 70 °C. After cooling, the mixture was filtered with Whatman paper no. 1 and centrifuged at 10 000 rpm for 10 minutes. The supernatant (extract) was collected and stored in dark bottles at 4 °C.

### Green synthesis of AuNPs using *C. tubulosa* extract

The gold nanoparticles were synthesized by mixing the extract and a 1 mM HAuCl_4_ solution in a 1 : 20 ratio. The pH of the mixture was adjusted to 10 using a 1 M NaOH solution, and the mixture was heated under reflux at 60 °C. The first characteristic of the formation of gold nanoparticles in the presence of the extract is the change in the solution's color from yellow to reddish-brown. Therefore, to select the best heating time to complete the gold nanoparticle synthesis reaction, the UV spectra of the solution were measured at various reaction times of 30, 40, 50, 60, 70, and 80 minutes. The optimum time was selected according to the peak appearing in the range of 500–600 nm. The solution was centrifuged at 12 000 rpm for 10 minutes. Finally, the remaining nanoparticles were washed three times with deionized water, and the adhesive fragments were separated and dried at room temperature in the dark for 36 h.

### Characterizations

A UV/vis spectrophotometer (Lambda 35, PerkinElmer) was used to select the optimum heating time to complete the synthesis reaction of AuNPs through evaluate the absorption peak appeared at a wavelength of 545 nm at various times. The size and morphology of AuNPs were investigated using a field emission scanning electron microscope (FESEM) (Sigma VP, ZEISS Co.) equipped with energy dispersive X-ray spectroscopy (EDX) and a transmission electron microscope (TEM) (EM10C-100KV-ZEISS). A ZetaSizer analyzer (Nano ZS, Malvern Instruments) was applied to measure their zeta potential and study the presence of charged bioactive compounds. The presence of different functional groups in the aqueous extract of *C. tubulosa* and their role in the reduction and stabilization of the formed AuNPs were evaluated using a Fourier transform infrared (FTIR) device (SPECTRUM 2, PerkinElmer). The crystalline nature of the synthesized AuNPs was determined by an X-ray diffractometer (XRD) (XPert Pro, Panalytical Co.).

#### Antibacterial assays

Antibacterial activities were determined using the disk-diffusion method according to the criteria set by the Clinical & Laboratory Standards Institute (CLSI) M100.^33^ The used microorganisms included *Pseudomonas aeruginosa* (ATCC27853) and *Escherichia coli* (ATCC25922) as the Gram-negative bacteria, and *Staphylococcus aureus* (ATCC25923) and *Bacillus cereus* (ATCC6633) as the Gram-positive bacteria, which were purchased from the Pasteur Institute of Tehran, Iran. After culturing the bacteria, a suspension was prepared from the bacterial strains. Then, 450 µg per mL AuNPs, 1 g gentamicin as the positive control, and 10 mL water as the negative control were added. Antibacterial activities were measured by the serial dilution method in a 96-well microtiter plate. Finally, minimum inhibitory concentration (MIC), minimum bactericidal concentration (MBC), and zone of inhibition (ZOI) were measured, and the data were statistically analyzed.

#### Antiparasitic assays

To investigate the antiparasitic activity of the synthesized AuNPs, *Giardia lamblia* cysts were collected from samples of excreted stools. These stools were obtained from patients by Shahid Ashrafi Isfahani Laboratory (Yasuj, Iran) for the purpose of clinical diagnosis. What remained of each sample was anonymized and used within this study. 10 g of stool was mixed with 50 mL of physiological saline and stirred for 30 min. The resulting solution was filtered through a 4-layer filter. The samples were centrifuged at 800 rpm for 5 min. After pouring the supernatant, 20 mL sucrose 2 M was added to the test tubes and centrifuged at 1800 rpm for 10 min. The supernatant containing cysts was separated, and 10 mL of normal saline (0.9%) was added to it. The solution was centrifuged at 1000 rpm for 5 min. Anti-giardial activities were measured using different concentrations of AuNPs (15, 30, 60, 120, and 240 µg mL^−1^) at various times (30, 60, 120, 240, and 480 min). Cysts were counted using light microscopy, and the percentage of *Giardia lamblia* cysts was reported.^34^

#### Scolicidal assays

To investigate the scolicidal activity of the synthesized gold nanoparticles, several sheep livers containing hydatid cysts were obtained from a local slaughterhouse and transported to the laboratory under appropriate conditions. After initial washing of the liver with physiological serum, in a sterile environment, the cysts were separated from the liver and disinfected with 70% ethanol. The protoscolices in the cysts were extracted and suctioned with a syringe with an 18-gauge needle and washed three times with physiological serum. The protoscolices were stored in dark containers containing normal saline and at a temperature of 4 °C scolicidal activity was performed with a drop of solution containing at least 500 protoscolices exposed to positive control (saturated saline), negative control (physiological serum) and different concentrations of biosynthesized gold nanoparticles from *C. tubulosa* (15, 30, 60, 90, and 180 µg mL^−1^) at time points of 5, 10, 15, 30, and 60 min. The mortality rates were determined by counting dead protoscolices using 0.1% vital eosin staining.

#### Statistical analysis

For statistical analysis of antibacterial, anti-giardia, and scolicidal activity data, after evaluating the normality of the data using the Kolmogorov–Smirnov test, the homogeneity of variances was studied using Bartlett's test. Then, the data were analyzed using the factorial test method based on the completely randomized design by SPSS Statistics 26.0 in three replications. For all analyses, the mean data were compared using Duncan's multiple range test at a 5% probability level, and their graphs were drawn.

## Results and discussion

**Fig. 1:**
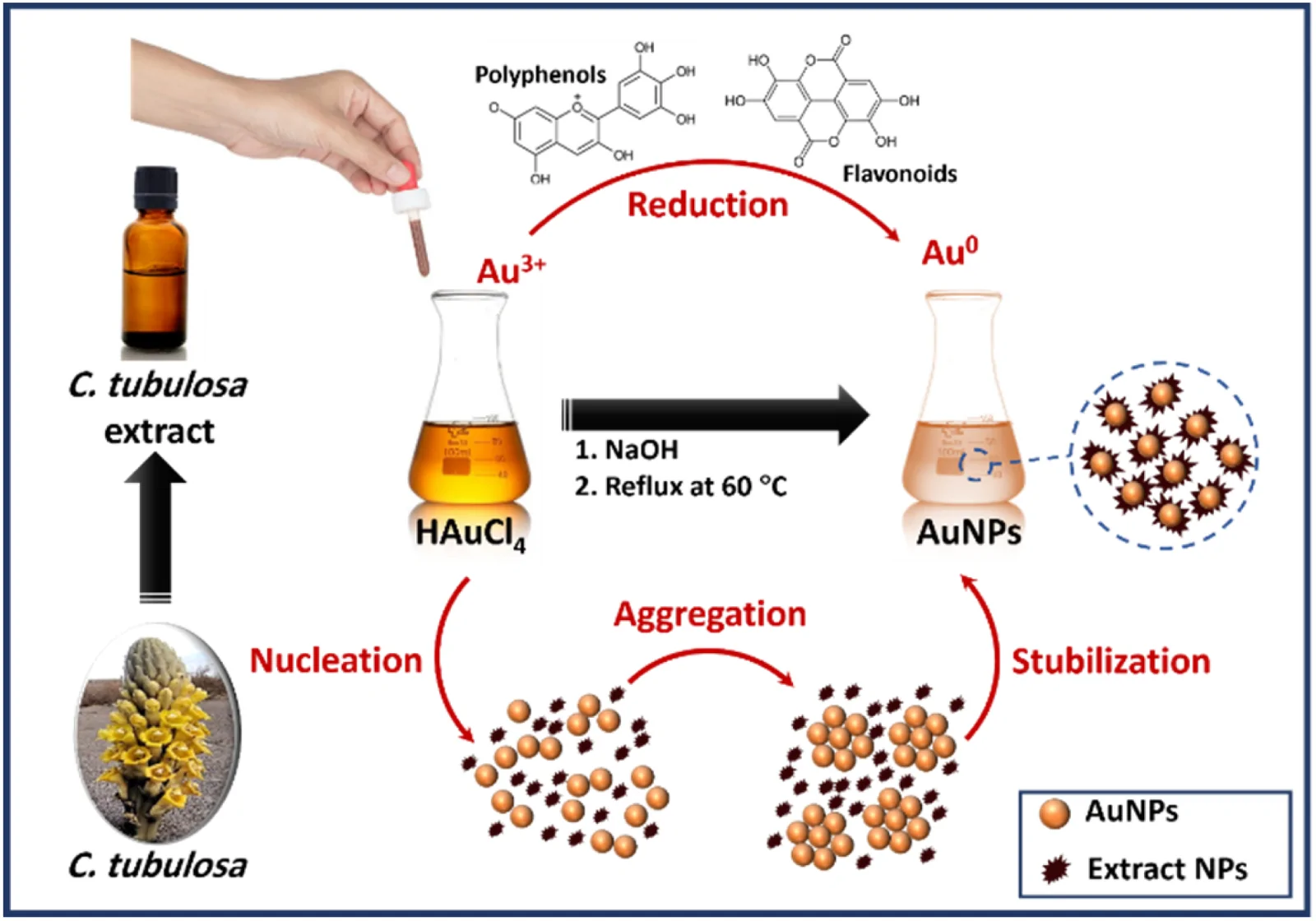
A schematic of synthesize AuNPs in presence of *C. tubulosa* extract and the mechanism of AuNPs synthesis and stabilization.

In this work, a green synthesis process is presented for the biosynthesis of AuNPs using *C. tubulosa* extract as an eco-friendly reducing/stabilizing two-functional agent. Fig. 1 displays a schematic of synthesize AuNPs in presence of *C. tubulosa* extract and the mechanism of AuNPs synthesis and stabilization. The important role of *C. tubulosa* extract in the biosynthesis of AuNPs is its reduction capability. The present phytochemicals in the structure of *C. tubulosa* extract, such as flavonoids, polyphenols, and amino acids, can reduce the gold ions (Au^3+^) to neutral gold (Au^0^). Also, it can play a useful role in stabilizing the synthesized AuNPs. The reduced gold atoms begin to aggregate, forming small clusters or nuclei. These nuclei grow further through further reduction of gold ions and incorporation into the structure. *C. tubulosa* extract acts as a capping or stabilizing agent in this stage. They surround the AuNPs, preventing them from aggregating into larger particles and stabilizing their size and shape.

**Fig. 2:**
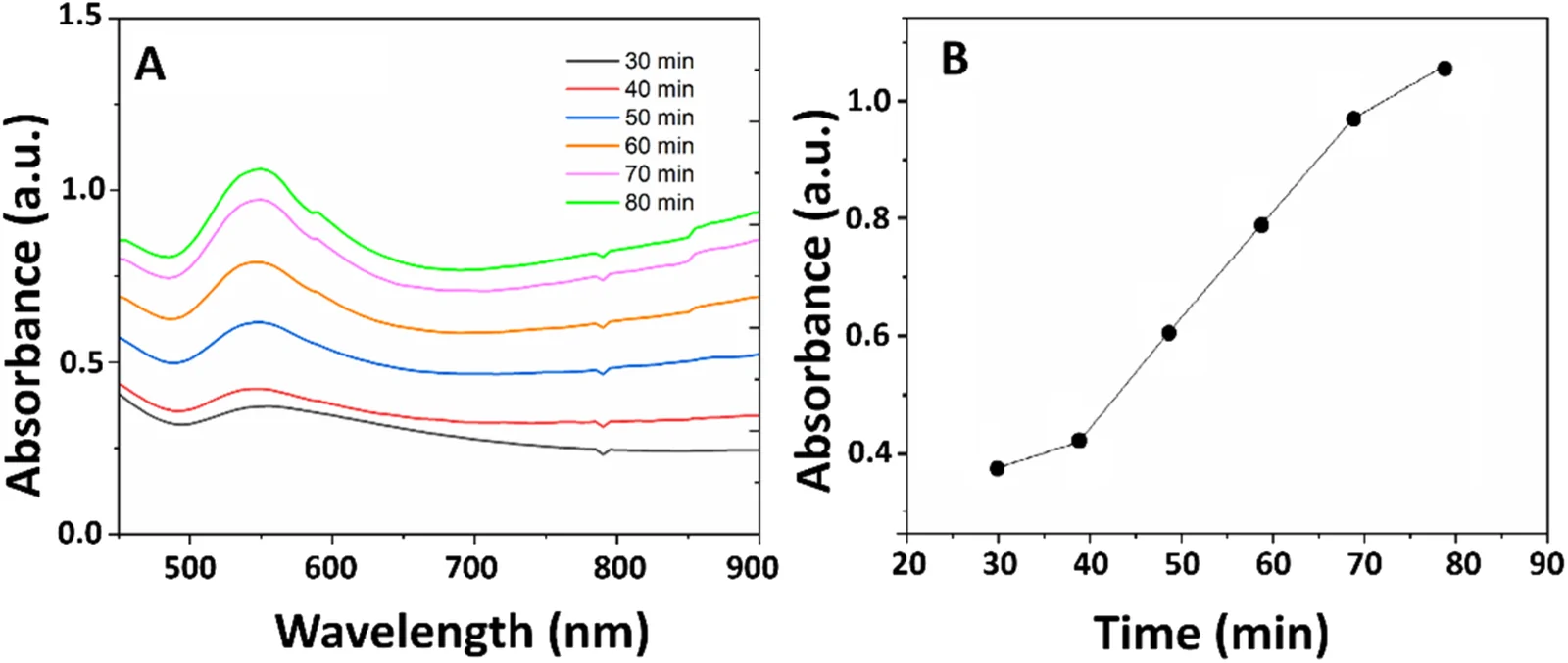
(A) UV-vis spectra of gold nanoparticles synthesized by *C. tubulosa* extract at different times, (B) the time-dependent kinetics of gold nanoparticle synthesis.

To investigate the biosynthesis of gold nanoparticles in the presence of the extract, UV-vis spectra were recorded and analyzed at reaction times of 30, 40, 50, 60, 70, and 80 min. As seen in Fig. 2A, a significant absorption peak appeared at a wavelength of 545 nm, whose intensity increased with increasing reaction time from 30 to 80 minutes. The increase in the intensity of the nanometer absorption peak and also the change in the color of the solution to dark brown are due to the excitation of surface plasmon resonance, which indicates the reduction of gold ions to gold nanoparticles.

The time-dependent kinetics of gold nanoparticle synthesis by the extract were investigated by plotting the changes in the absorption peak at 545 nm with time. Fig. 2B shows that the absorption intensity increased at a significant rate in the early stages of nanoparticle synthesis, indicating the formation of gold nanoparticles within 30 to 70 minutes. After 70 minutes, the rate of increase in peak intensity decreased, indicating the completion of the gold nanoparticle formation reaction.

### Characterizations

#### Size and morphology

**Fig. 3:**
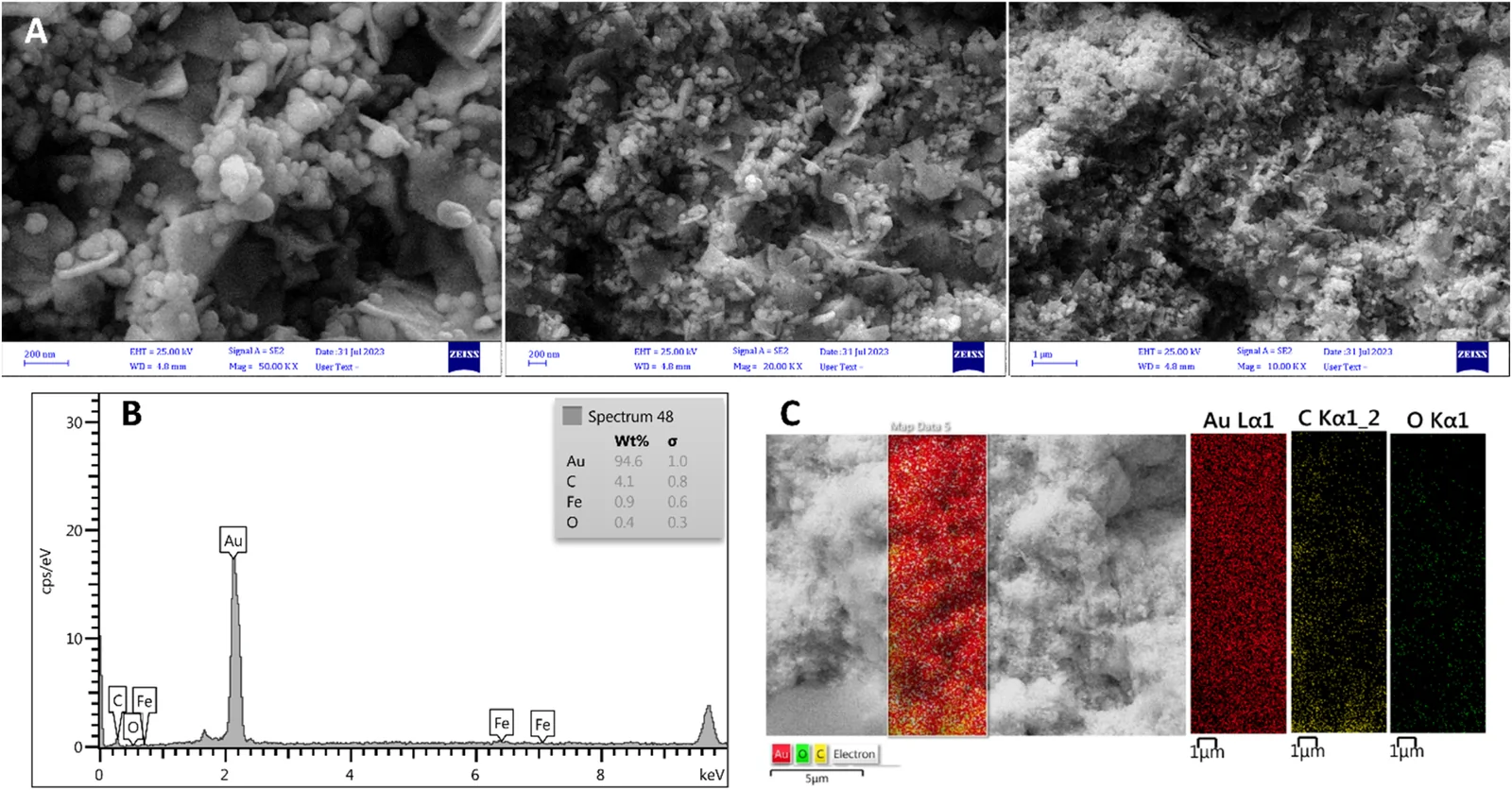
(A) FESEM images with different magnifications, (B) EDX analysis, and (C) mapping pattern of the synthesized AuNPs in presence of *C. tubulosa* extract.

FESEM images of the synthesized AuNPs in the presence of *C. tubulosa* extract are indicated in Fig. 3A. As can be seen, AuNPs were successfully synthesized with quasi-spherical morphology in the particle size range of 20–70 nm. To identify the present elements in the synthesized gold nanoparticles, EDX analysis and mapping pattern were used, the results of which are shown in Fig. 3B and C. In addition to gold (94.6%), which was the dominant constituent element of the synthesized nanoparticles, oxygen (4.1%) and carbon (1.4%) elements related to the compounds present in *C. tubulosa* extract were also present in EDX analysis, which play a reducing and stabilizing role in the synthesis of gold nanoparticles. The mapping pattern also indicates a uniform distribution of gold throughout the synthesized nanoparticles.

**Fig. 4:**
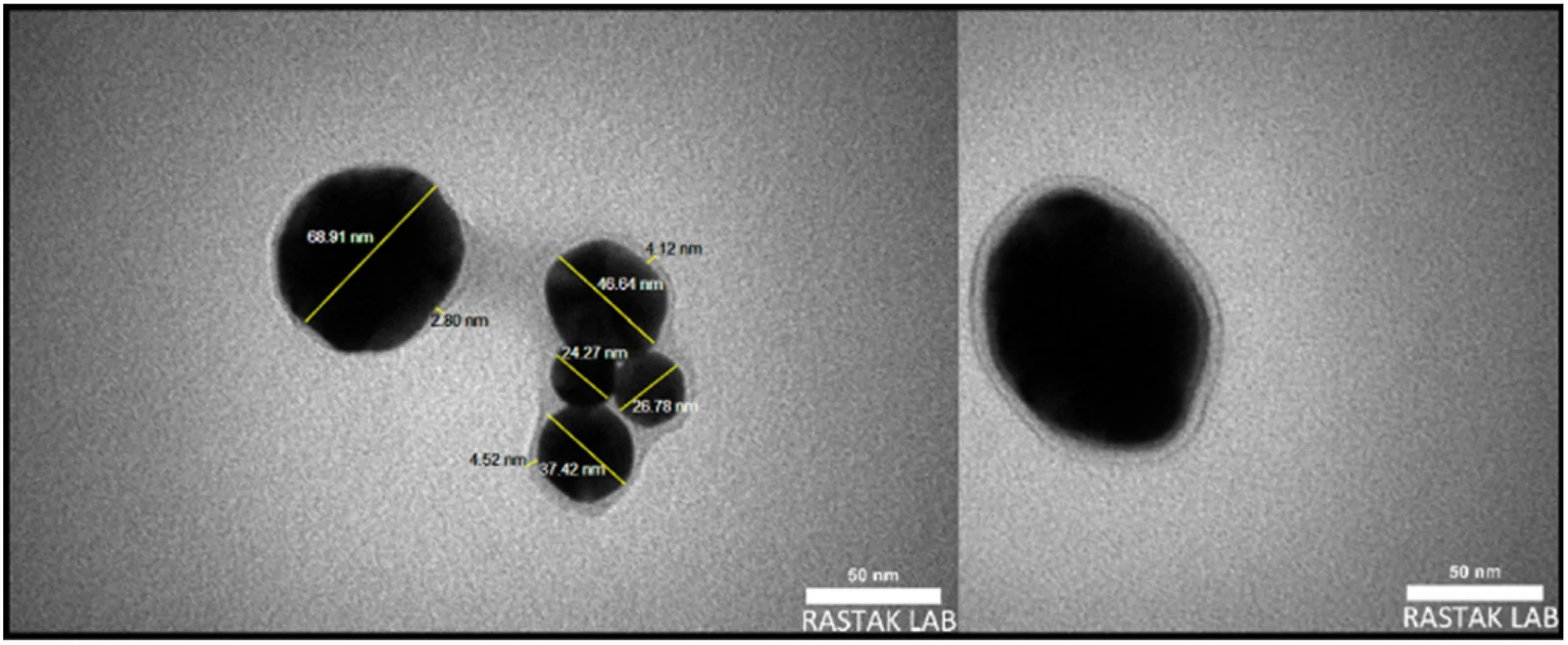
TEM images of the synthesized AuNPs in presence of *C. tubulosa* extract.

The morphology, size, and aggregation of biosynthesized AuNPs in the presence of *C. tubulosa* extract were more clearly investigated by TEM. As can be seen in Fig. 4, gold nanoparticles with spherical morphology and dark color are visible. According to this figure, gold nanoparticles have dimensions of about 20 to 70 nm, which is consistent with FESEM images. Also, a layer with dimensions of less than 5 nm is observed on the surface of nanoparticles, which can be attributed to *C. tubulosa* extract as the capping agent, which causes the surface of nanoparticles to be charged and, while increasing the absolute value of zeta potential, leads to the creation of repulsive forces between particles and therefore their stability in colloids.

**Fig. 5:**
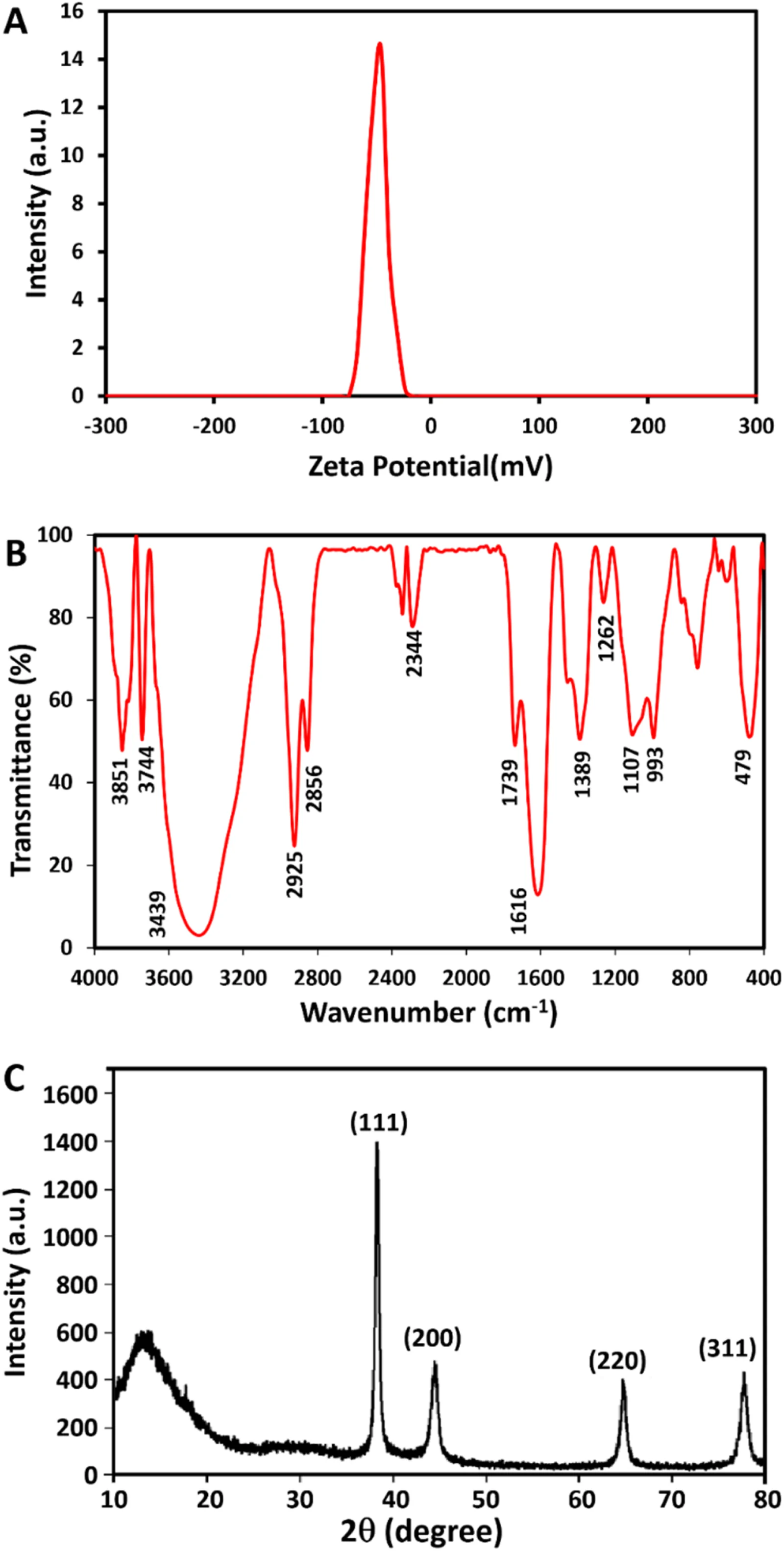
(A) Zeta potential, (B) FT-IR spectrum, and (C) XRD pattern of the synthesized AuNPs.

The zeta potential test was used to measure the colloidal stability and surface charge of AuNPs produced from *C. tubulosa* (Fig. 5A). The zeta potential value of the sample at the maximum point was −48.9 mV, and the electrophoretic mobility of the particles was obtained as −3.78 µm cm V^−1^ s^−1^. The negative value of the zeta potential describes the presence of functional groups and the presence of negatively charged bioactive compounds, including phenolic, polyphenolic, and total phenolic compounds, on the surface of the AuNPs, which provides the stabilization and efficient biosynthesis of AuNPs using *C. tubulosa* extract. In general, the tendency of like-charged particles to repel each other has a direct relationship with the zeta potential, so that particles with an absolute value of zeta potential greater than 30 mV are relatively stable in the colloidal medium.

#### Structure and crystallinity

To investigate the functional groups and chemical bonds present in the synthesized gold nanoparticles in the presence of *C. tubulosa* extract, which play a reducing and stabilizing role in the synthesis of gold nanoparticles, the FT-IR spectrum of AUNPs was investigated. Fig. 5B shows the FT-IR spectrum of the synthesized AuNPs. The most important peak appearing in the spectrum is related to the stretching vibration of gold nanoparticles, which appeared at 479 cm^−1^.^35^ The peaks appearing at 3851 cm^−1^, 3744 cm^−1^, and 3439 cm^−1^ are related to the asymmetric and symmetric stretching vibrations of O–H bonds in aromatic and aliphatic structures (phenols, terpenoids, and saponins) in *C. tubulosa* extract.^36,37^ The peaks related to the asymmetric and symmetric stretching vibration of the C–H bond in the methyl and methylene structures appeared at 2925 cm^−1^ and 2856 cm^−1^, respectively. The peaks related to the stretching vibration of the C=O bond in the carbonyl group and the stretching vibration of the C=C bonds in the aromatic rings in the structure of phenylethanoid glycosides and lignans in *C. tubulosa* extract appear at 1739 cm^−1^ and 1616 cm^−1^, respectively. The peak at 1389 cm^−1^ is related to the bending vibration of the C–H bond in the structure of the various compounds in this extract, respectively. The peaks at 1107 cm^−1^ and 993 cm^−1^ are related to the stretching vibrations of the C–OH and C–O–C bonds, respectively, and the peak located at 1757 cm^−1^ is related to the out-of-plane vibration of the C–H bond attached to the aromatic rings.^38,39^

XRD assay was performed to investigate the crystalline properties of nanoparticles synthesized using *C. tubulosa* extract, the pattern of which is shown in Fig. 5C. The peaks appearing at 2*θ* = 3.38°, 5.44°, 64°, and 7.77° are related to reflections of (111), (200), (220), and (311), which are identical with the standard pattern for the crystal structure of gold by Joint Committee on Powder Diffraction Standards (JCPDS) no. 04-0784. This indicates the successful synthesis of gold nanoparticles with a spherical crystal structure.^40,41^ The appearance of a broad peak in the range of 10° to 25° can be attributed to the presence of organic groups present in the extract with an amorphous structure on the surface of nanoparticles, which are responsible for preventing the nanoparticles from being adsorbed to each other in the colloid.^42,43^

#### Antibacterial assay

The antibacterial activity of green synthesized gold nanoparticles using *C. tubulosa* extract at a concentration of 450 µg mL^−1^ was investigated against four bacteria of *P. aeruginosa*, *E. coli*, *S.aureus* and *B. cereus* and compared with the antibiotic gentamicin. As presented in Table 1, the synthesized gold nanoparticles at lower concentrations have the maximum inhibitory effect on the Gram-positive bacteria with MIC of 37.50 ± 16.23 µg mL^−1^ for *B. cereus* and MIC of 75.00 ± 32.47 µg mL^−1^ for *S. aureus*, while the two Gram-negative bacteria showed greater resistance to the nanoparticles. The results of MBC evaluation show that Gram-positive bacteria *B. cereus* with MBC of 225.00 ± 0.00 µg mL^−1^ and *S. aureus* with MBC of 300 ± 129.90 µg mL^−1^ were killed at lower concentrations of *C. tubulosa* extract, but to kill Gram-negative bacteria *P. aeruginosa* and *E. coli*, a higher concentration (450 µg mL^−1^) was required, indicating their higher resistance to biosynthesized nanoparticles. The results related to ZOI indicate that the highest antimicrobial activity of synthesized gold nanoparticles was on *B. cereus* with ZOI of 19.20 ± 0.26 mm, followed by *S. aureus*, *E. coli*, and *P. aeruginosa*. Analysis of the mean MIC, MBC, and ZOI indicates that the synthesized gold nanoparticles in the presence of *C. tubulosa* extract had a significant antibacterial effect (*P* < 0.01) on all four Gram-positive and Gram-negative bacteria compared to gentamicin as the positive control.

**Table 1:** Antibacterial activity of AuNPs biosynthesized by *C. tubulosa* extract^a^

Bacteria	Mean ± standard deviation of mortality rate						Negative ctrl		
	AuNPs (450 µg mL^−1^)			Gentamicin (64 µg mL^−1^) positive ctrl					
	MIC	MBC	ZOI	MIC	MBC	ZOI	MIC	MBC	ZOI
*P. aeruginosa*	225 ± 0.00^a^	450 ± 0.00^a^	12.23 ± 0.25^g^	13.33 ± 4.62^c^	32.00 ± 0.00^d^	19.50 ± 0.30^d^	N.S	N.S	N.S
*E. coli*	187.50 ± 64.95^a^	375 ± 129.90^ab^	13.10 ± 0.26^f^	10.67 ± 4.62^c^	26.67 ± 9.24^d^	20.83 ± 0.72^c^	N.S	N.S	N.S
*S.aureus*	75.00 ± 32.47^b^	300 ± 129.90^bc^	16.76 ± 0.71^e^	5.33 ± 2.31^c^	10.67 ± 4.62^d^	23.13 ± 0.30^b^	N.S	N.S	N.S
*B. cereus*	37.50 ± 16.23^bc^	225 ± 0.00^c^	19.20 ± 0.26^d^	4.00 ± 0.00^c^	8.00 ± 0.00^d^	24.07 ± 0.42^a^	N.S	N.S	N.S

a MIC: Minimum inhibitory concentration. MBC: minimum bactericidal concentration. ZOI: zone of inhibition. NS: non-sensitive. Different letters within each column of MIC, MBC, and ZOI indicate statistically significant differences (*P* value ≤ 0.05).

Most of the antibacterial activity of the synthesized gold nanoparticles can be attributed to the absence of an outer membrane in their cell wall. Various mechanisms have been proposed for the antibacterial activity of gold nanoparticles. The first mechanism is the disruption of cellular processes through cellular respiration when gold nanoparticles come into contact with bacteria and bind to the surface of the bacterial cell membrane. According to another mechanism, when metal ions, especially gold, come into contact with bacteria and disperse in the environment around the bacterial cell, a concentrated source of metal ions is formed, which causes the release of ions and increases cytotoxicity.^44,45^ The mechanism of antibacterial activity of gold nanoparticles synthesized with plant extracts occurs in two stages. First, the nanoparticles cause changes in the membrane potential and then reduce the activity of ATP synthase. In the second stage, the nanoparticles prevent the binding of the ribosomal subunit to tRNA, which has a great impact on the destruction of the biological processes of the bacterial cell.^46,47^

#### Antiparasitic assay

**Fig. 6:**
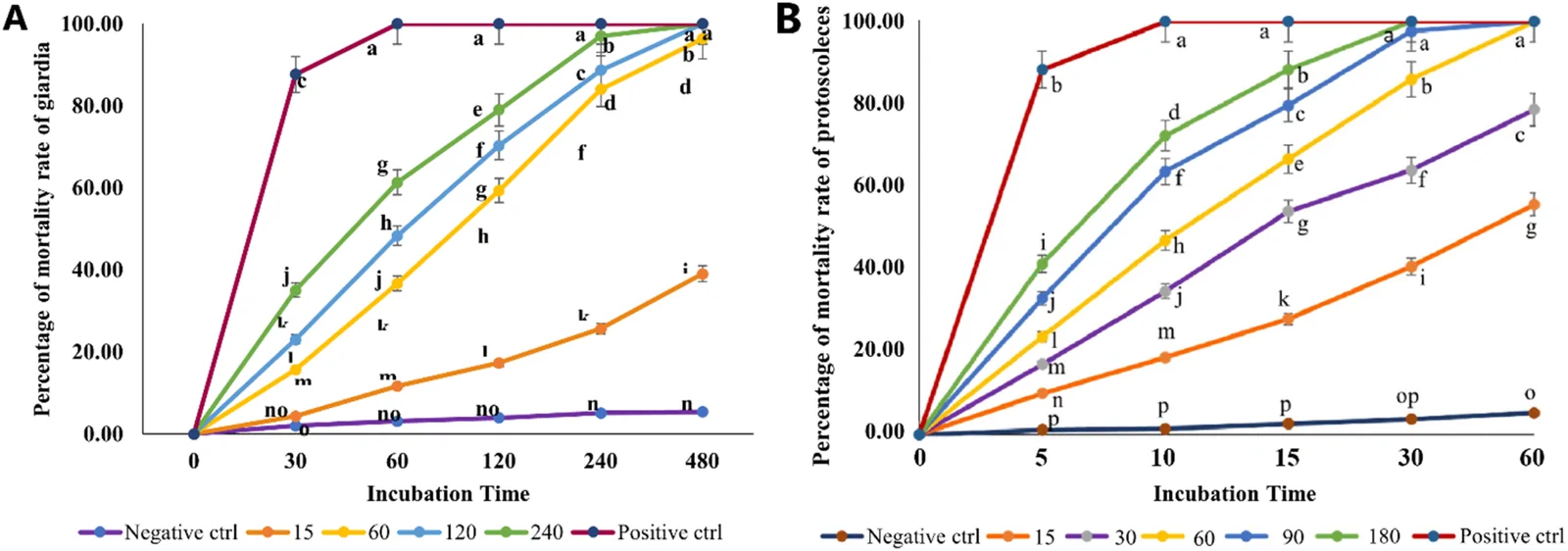
(A) Antiparasitic activity, and (B) anti-protoscolicidal activity of the biosynthesized gold nanoparticles using different concentrations and times.

The antiparasitic activity of biosynthesized gold nanoparticles in the presence of *C. tubulosa* extract was evaluated using different concentrations of 15, 30, 60, 120, and 240 µg mL^−1^ at various times of 30, 60, 120, 240, and 480 min. As can be seen in Table 2 and Fig. 6a, more than 96% of *Giardia lamblia* cysts were killed within 480 minutes in the presence of gold nanoparticles at a concentration of 60 µg mL^−1^ and 100% of cysts were killed within 480 minutes in the presence of gold nanoparticles at a concentration of 120 µg mL^−1^ or more. Also, the lethal effect of gold nanoparticles at concentrations of 120 and 480 µg mL^−1^ and a time of 480 minutes was consistent with the effect of metronidazole as the drug of choice in the treatment of *Giardia lamblia* at times of 60 minutes onwards (*P* > 0.05). Therefore, the effect of synthesized gold nanoparticles in the presence of the extract on the *Giardia lamblia* parasite was dose-dependent, which increased with time (*P* ≤ 0.001). The antiparasitic activity of gold nanoparticles is through inhibition and destruction of cell walls, inhibition of protein and DNA synthesis. Our proposed mechanism is based on damage to the parasite through interference of gold nanoparticles with its DNA, which leads to abnormalities in cell division, stimulation of the excretion of reactive oxygen species, induction of oxidative stress in cells, and ultimately its death.^48,49^

**Table 2:** Antiparasitic activity of AuNPs biosynthesized by *C. tubulosa* extract^a^

Mean ± standard deviation of mortality rate of giardia (%)							
Concentration → incubation time↓	15 µg mL^−1^	30 µg mL^−1^	60 µg mL^−1^	120 µg mL^−1^	240 µg mL^−1^	Negative control	Positive control
30 Min	4.33 ± 1.15^no^	10.33 ± 0.57^m^	15.67 ± 1.15^l^	23.00 ± 1^k^	35.00 ± 3^j^	2.01 ± 0.13^o^	87.67 ± 0.57^c^
60 Min	11.67 ± 1.01^m^	24.00 ± 1^k^	36.67 ± 2.08^j^	48.33 ± 2.88^h^	61.33 ± 2.31^g^	3.10 ± 0.86^no^	100 ± 0^a^
120 Min	17.33 ± 1.52^l^	46.33 ± 2.31^h^	59.33 ± 4.04^g^	70.33 ± 3.21^f^	79.00 ± 1^e^	3.93 ± 0.28^no^	100 ± 0^a^
240 Min	25.67 ± 2.08^k^	69.67 ± 2.08^f^	84.00 ± 0^d^	88.67 ± 0.58^c^	97.00 ± 1.73^b^	5.10 ± 0.10^n^	100 ± 0^a^
480 Min	39.00 ± 1^i^	83.33 ± 1.52^d^	96.33 ± 2.31^b^	100.00 ± 0^a^	100.00 ± 0^a^	5.40 ± 0.28^n^	100 ± 0^a^

a Different letters indicate statistically significant differences (*P* value ≤ 0.05).

#### Scolicidal assay

The anti-protoscolicidal activity of biosynthesized gold nanoparticles was evaluated using different concentrations (15, 30, 60, 90, and 180 µg mL^−1^) at various times (5, 10, 15, and 30 min) on hydatid cyst protoscolices. Table 3 and Fig. 6b show the results of this evaluation. The highest scolicidal activities of biosynthesized gold nanoparticles in the presence of *C. tubulosa* extract were obtained using a concentration of 180 µg mL^−1^ at 30 min and concentrations of 60 and 90 µg mL^−1^ at 60 minutes, and the lowest scolicidal activity (10%) was observed at a concentration of 15 µg mL^−1^ of gold nanoparticles at 5 minutes (*P* ≤ 0.001). Thus, the activity of nanoparticles on liver hydatid cyst protoscolices was dose-dependent, and this effect also increased significantly with time (*P* ≤ 0.001). These differences in lethality are likely to depend on factors such as the type of plant used, the synthesis method of gold nanoparticles, time, and concentration. The proposed mechanism of anti-protoscolicidal activity of gold nanoparticles is based on the entry of nanoparticles into their cells and the disruption of the function of enzymes present in the cell.^50^ Comparing the anti-protoscolicidal activity of synthesized gold nanoparticles with other studies shows better performance of gold nanoparticles prepared in the presence of *C. tubulosa* extract, as they were able to eliminate 100% of hydatid cyst protoscolices at lower concentrations and in a shorter time.^51–53^

**Table 3:** Anti-protoscolicidal activity of various concentrations of biosynthesized AuNPs at different times^a^

Mean ± standard deviation of mortality rate of protoscoleces (%)							
Concentration → incubation time↓	15 µg mL^−1^	30 µg mL^−1^	60 µg mL^−1^	90 µg mL^−1^	180 µg mL^−1^	Negative control	Positive control
5 Min	10± 0^n^	17 ± 1.73^m^	23.67 ± 1.53^l^	33 ± 3^j^	41.33 ± 2.31^i^	1.15 ± 0.13^p^	88.33 ± 0.57^b^
10 Min	18.67 ± 1.53^m^	34.67 ± 0.58^j^	47 ± 1.73^h^	63.67 ± 1.53^f^	72.33 ± 2.52^d^	1.47 ± 0.12^p^	100 ± 0^a^
15 Min	28±2^k^	54 ± 1.73^g^	66.67 ± 3.21^e^	79.67 ± 0.58^c^	88.33 ± 0.58^b^	2.65 ± 0.23^p^	100 ± 0^a^
30 Min	40.67 ± 1.15^i^	64 ± 1.73^f^	86 ± 1.73^b^	97.67 ± 2.51^a^	100 ± 0^a^	3.78 ± 0.39^op^	100 ± 0^a^
60 Min	55.67 ± 3.05^g^	78.67 ± 2.52^c^	100 ± 0^a^	100 ± 0^a^	100 ± 0^a^	5.3 ± 0.33^o^	100 ± 0^a^

a Different letters indicate statistically significant differences (*P* value ≤ 0.05).

## Conclusions

This study successfully demonstrates the green synthesis of gold nanoparticles using *C. tubulosa* extract, highlighting their significant antibacterial, antiparasitic, and scolicidal properties. It is worth noting that recent studies on *Cistanche* species indicated that their crude extract typically does not exhibit a high antibacterial activity. For instance, Abdallah (2017) and Djemouaı *et al.* (2024) demonstrated modest antimicrobial activity in extracts of *Cistanche violacea*.^54,55^ Therefore, it can be concluded that the strong bioactivity of the synthesized AuNPs is due to the synergistic effect of the biological properties of the *C. tubulosa* extract and gold nanoparticles. The synthesized AuNPs offer a promising alternative to conventional antimicrobial agents, with high efficacy against both bacterial pathogens and parasitic diseases, and are a valuable candidate for further development in therapeutic applications. Although the green and phytochemical-based synthesis route indicates the potential biocompatibility of the obtained AuNPs, the current study did not incorporate assessments of cytotoxicity or cell viability. Consequently, the safety profile of these nanoparticles necessitates validation through targeted *in vitro* cytotoxicity assays and *in vivo* evaluations in future research. Also, future studies should focus on optimizing the synthesis conditions and exploring the *in vivo* effectiveness of these nanoparticles in clinical settings to fully realize their potential as novel therapeutic agents for treating infectious and parasitic diseases.

## Ethical statement

Ethical approval for this study was granted by the Research Ethics Committee of Yazd University (Approval ID: IR.YAZD.REC.1401.076). The research did not involve any experiments with live animals or direct interaction with human subjects. Sheep livers containing hydatid cysts were procured from a local slaughterhouse, while the human stool samples utilized for Giardia isolation were anonymized remnants of specimens previously collected for routine clinical diagnosis.

## Author contributions

Hanieh Karimi: experiment, writing – original draft, software, methodology, analysis. S. Ebrahim Seifati: supervision, conceptualization, writing – review & editing. Damoun Razmjoue: validation, methodology, resources. Soraya Ghayempour: writing – review & editing, conceptualization, validation.

## Conflicts of interest

There are no conflicts to declare.

## Data Availability

All data have been included in main text of article and no new data were generated or analysed as part of this article.
